# Pediatric malignant pheochromocytoma with atypical presentation as vision changes, lung metastasis, and recurrence: a case report

**DOI:** 10.1186/s13256-023-04329-7

**Published:** 2024-03-05

**Authors:** Kaijun Zhang, Penghui Yang, Mi Li, Ping Xiang, Zhenli Cheng, Xue Zhou

**Affiliations:** Department of Cardiovascular Medicine, Children’s Hospital of Chongqing Medical University, National Clinical Research Center for Child Health and Disorders, Ministry of Education Key Laboratory of Child Development and Disorders, Chongqing Key Laboratory of Pediatrics, Zhongshan 2nd Road, No.136, Yuzhong District, Chongqing, 400014 China

**Keywords:** *SDHB* mutation, Malignant pheochromocytoma, Vision changes, Case report

## Abstract

**Background:**

This case report documents a case of malignant pheochromocytoma manifested as vision changes with lung metastasis and recurrence.

**Case presentation:**

A 10-year-old Han Chinese girl presented with vision changes and was eventually diagnosed with pheochromocytoma by contrast-enhanced computed tomography, urine vanillylmandelic acid. After medication for hypertension and surgery, clinical symptoms disappeared. Malignant pheochromocytoma with lung metastasis was confirmed histologically using the Pheochromocytoma of the Adrenal Gland Scaled Score scoring system and genetically with *succinate dehydrogenase complex iron sulfur subunit B* mutation, and 3 months later, unplanned surgery was performed because of the high risks and signs of recurrence. She is asymptomatic as of the writing of this case report. Our patient’s case highlights the importance of considering a diagnosis of malignant pheochromocytoma, and long-term follow-up for possible recurrence.

**Conclusion:**

Although there are well-recognized classic clinical manifestations associated with pheochromocytoma, atypical presentation, such as vision changes in children, should be considered. In addition, malignant pheochromocytoma children with a high Pheochromocytoma of the Adrenal Gland Scaled Score and *succinate dehydrogenase complex iron sulfur subunit B* mutation require a long-term follow-up or even unplanned surgery because of the higher risk of recurrence.

**Supplementary Information:**

The online version contains supplementary material available at 10.1186/s13256-023-04329-7.

## Background

Pheochromocytoma, a rare and highly representative neuroendocrine tumor and one of the secondary factors of hypertension in children, is typically characterized by excessive release of catecholamines and high levels of circulating catechol, with most tumors being benign and having a good prognosis, while approximately 10% are malignant [1]. If left untreated, both benign and malignant pheochromocytoma can lead to severe or even fatal complications. However, diagnosing this “great masquerader” is challenging, especially vision changes, which are uncommon in children. Moreover, lung metastasis and recurrence were infrequently seen [2]. Herein, we described a very rare case of a girl with hypertension and vision changes who was eventually diagnosed with a case of malignant pheochromocytoma with lung metastasis and presented signs of recurrence.

### Case description

A 10-year-old Han Chinese girl was admitted to our cardiovascular department with significant left eye vision changes that had abruptly occurred 10 days prior. The changes were characterized by blurred vision, black spots, or shadows in the vision, with each episode lasting several days with spontaneous resolution. The patient reported poor eyesight history, heat intolerance, and fatigue following activity. No relevant family history was reported.

**Fig. 1:**
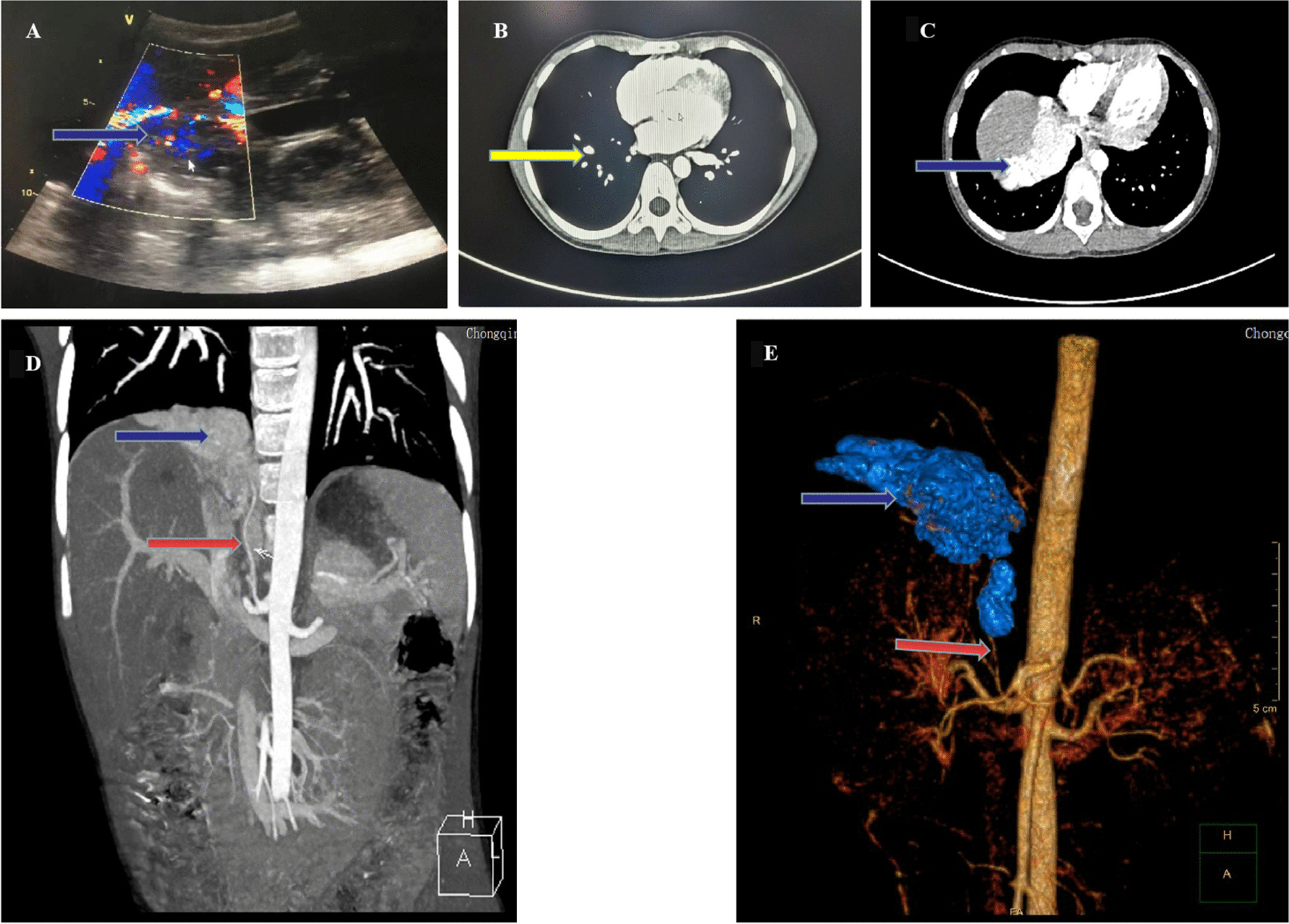
Abdominal ultrasound result showing that exploration and size of the right adrenal area was about 1.1 cm × 1 cm hypoechoic structure, with rich internal blood flow signal (blue arrow in **A**). Chest CT results showed many nodules in both lungs, and the largest nodule (yellow arrow in **B**) was located in the middle lobe of the right lung, with a size of about 8.2 mm × 7.0 mm. Abdominal CT results showed that the right adrenal gland was not clearly displayed and multiple enhanced nodules were found inside, with the largest having a size of 10.8 mm × 17.2 mm × 17.8 mm (blue arrow in **C**). CT angiography showed that the tumor lesion (blue arrows) was compressed adjacent to the inferior vena cava, and the right inferior adrenal artery had vascular branches (red arrows) into the tumor lesion (**D** and **E**)

On admission, fundus examination and fluorescein fundus angiography characterized hypertensive ocular funduscopic abnormalities (Additional file 1: Fig. S1). Further examinations were performed. Supine aldosterone assay was 54.3 (< 23.6) ng/dL, renin supine assay was 497.3 (2.8–39.9) uIU/mL, urine vanillylmandelic acid was 28.99 (0–13.60) mg/24 hours. Abdominal ultrasonography, contrast-enhanced computed tomography (CT) scan, and computed tomography angiography (CTA) showed space-occupying lesions with the possibility of malignant pheochromocytoma accompanied with lung metastasis (Fig. 1). Cortisol, adrenocorticotropic hormone, thyroid function, tumor biomarkers (carcinoembryonic antigen, alpha-fetoprotein, human chorionic gonadotropin, ferritin, neuron-specific enolase), autoantibodies, and antineutrophil cytoplasmic antibodies showed no obvious abnormal indicators. Echocardiography and electrocardiogram findings were normal.

**Fig. 2:**
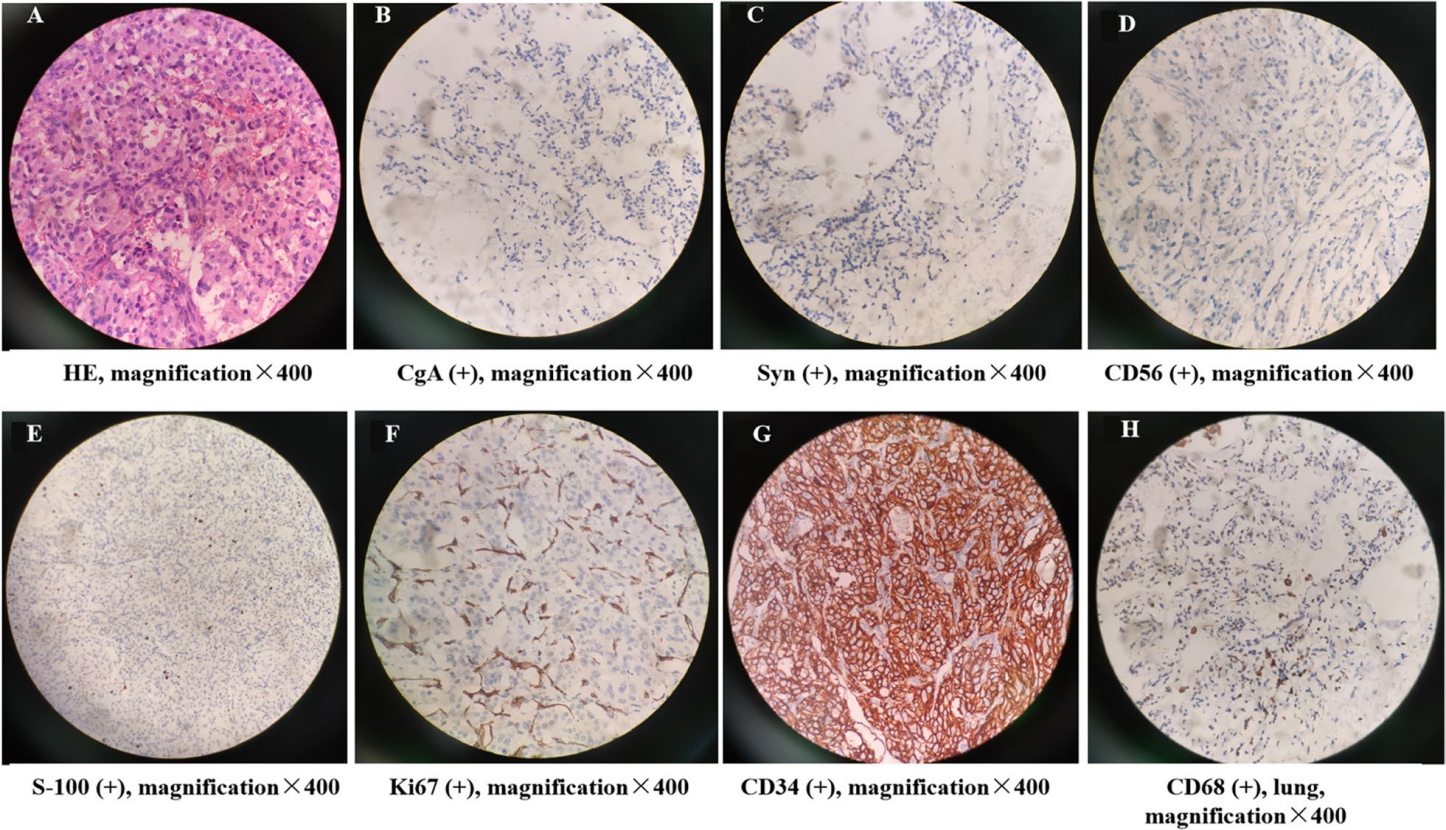
Tumor bigger than 6 cm in diameter with capsular invasion and extra-adrenal involvement, as seen by naked eye. By microscopy, large cell nests, increased cell density, spindle cells, tumor nuclear pleomorphism, and megakaryosis were found in **A** (hematoxylin and eosin, magnification, ×400). CgA (**B**), Syn (**C**), CD56 (**D**), S-100 (**E**), Ki67 (**F**), CD34 (**G**) antibody immunohistochemical showed positive reactivity in the tumor tissue; the positive rate of Ki67 staining was 2–10%; CD68 (**H**) antibody immunohistochemistry was positive in the lung tissue; magnification ×400

**Fig. 3:**
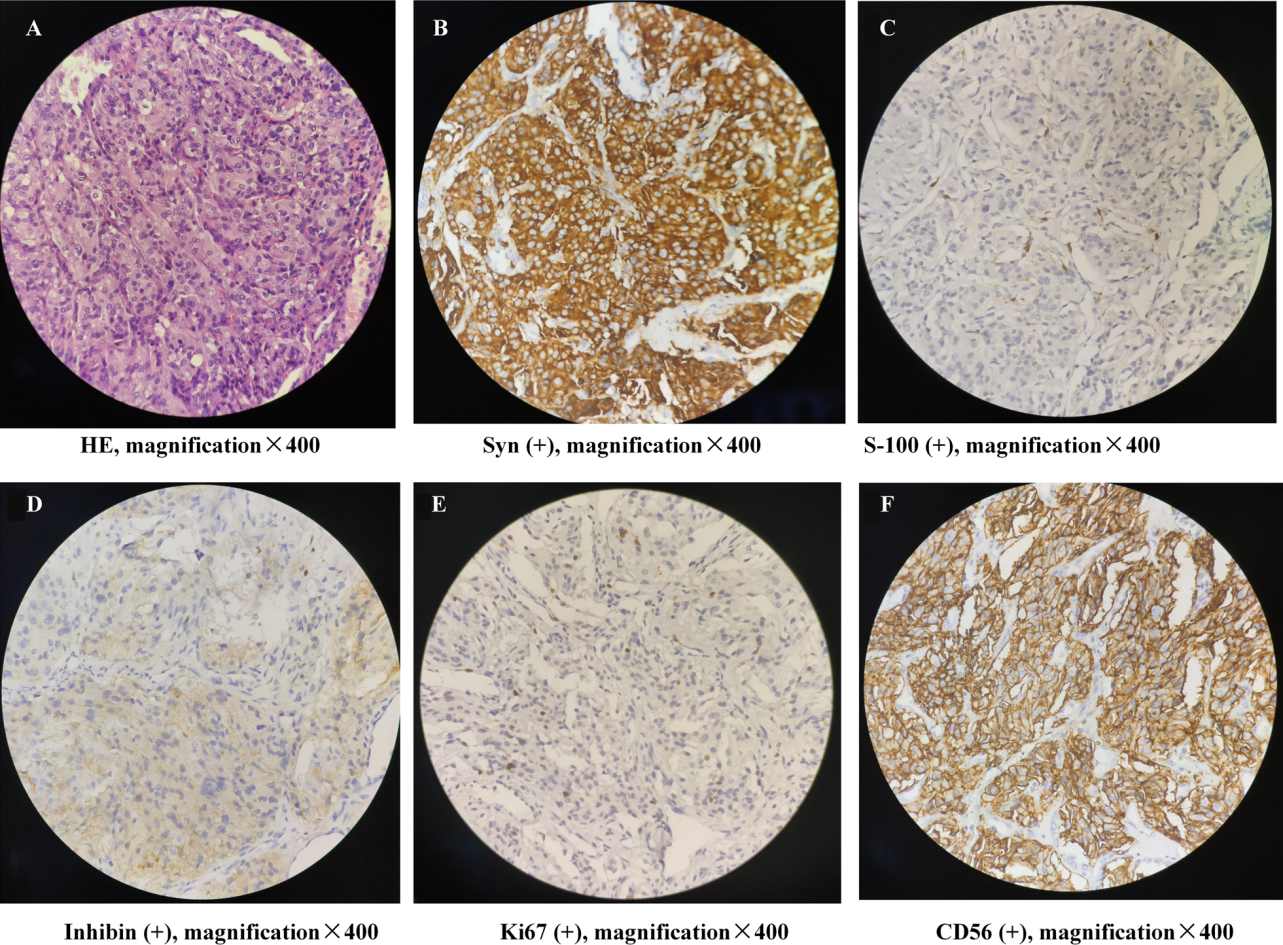
By microscopy, round or ovoid nuclei with marked pleomorphism, small foci of interstitial tumor cells, and giant nuclei found in **A** (hematoxylin and eosin, magnification, ×400). Syn (**B**), S-100 (**C**), inhibin (**D**), Ki67 (**E**), CD56 (**F**) antibody immunohistochemical showed positive reactivity in the lesion in the middle lobe of the lung; magnification ×400. Positive rate of Ki67 staining was 2–7%

Given the patient’s clinical manifestations, physical examination, laboratory tests, and imaging changes, preliminary diagnosis of pheochromocytoma was made. After the medication for hypertension (ɑ-blockers and calcium channel blockers), the right retroperitoneal mass, a portion of the right adrenal gland, and the suspected lesion in the middle lobe of the lung were surgically removed. Pathological results of the tumor and the lesion in the middle lobe of the lung were shown in Figs. 2 and 3, respectively. During the operation, infiltration of the tumor into part of the diaphragm and adrenal gland was observed. Pheochromocytoma of the Adrenal Gland Scaled Score (PASS) was used to differentiate benign (PASS < 4) from malignant (PASS ≥ 4) histologically, and this patient’s PASS score was at least 8 because of invasion [capsular (score = 1), periadrenal tissue (score = 2)], large nests or diffuse growth (score = 2), high cellularity (score = 2), and profound nuclear pleomorphism (score = 1) [3]. Consequently, malignant pheochromocytoma was verified. Finally, genetic testing revealed that *succinate dehydrogenase complex iron sulfur subunit B* (*SDHB*) heterozygous frameshift mutation in the exon 3/8 (c.237del, p.Lys80ArgfsTer8) was the possible pathogenic mutation sites related to tumor genetic susceptibility.

The urine vanillylmandelic acid, both aldosterone and renin in the supine position, had normalized after surgery, and the postoperative period before discharge was stable with obvious improvement of vision and significant decrease of blood pressure (Additional file 1: Fig. S2). Following discharge, the patient was completely asymptomatic, and abdominal ultrasound showed no obvious abnormality in the first week and the first month. During the latest follow-up at 3 months post-treatment, abdominal ultrasound showed right retroperitoneal parenchymal nodule with small calcified foci and blood supply. Given the high risk of recurrence both pathologically and genetically, an unplanned surgery to remove the possible recurring tissue was recommended. Thus far, the patient remains in good condition with no evidence of hypertension and great improvement in vision.

**Fig. 4:**
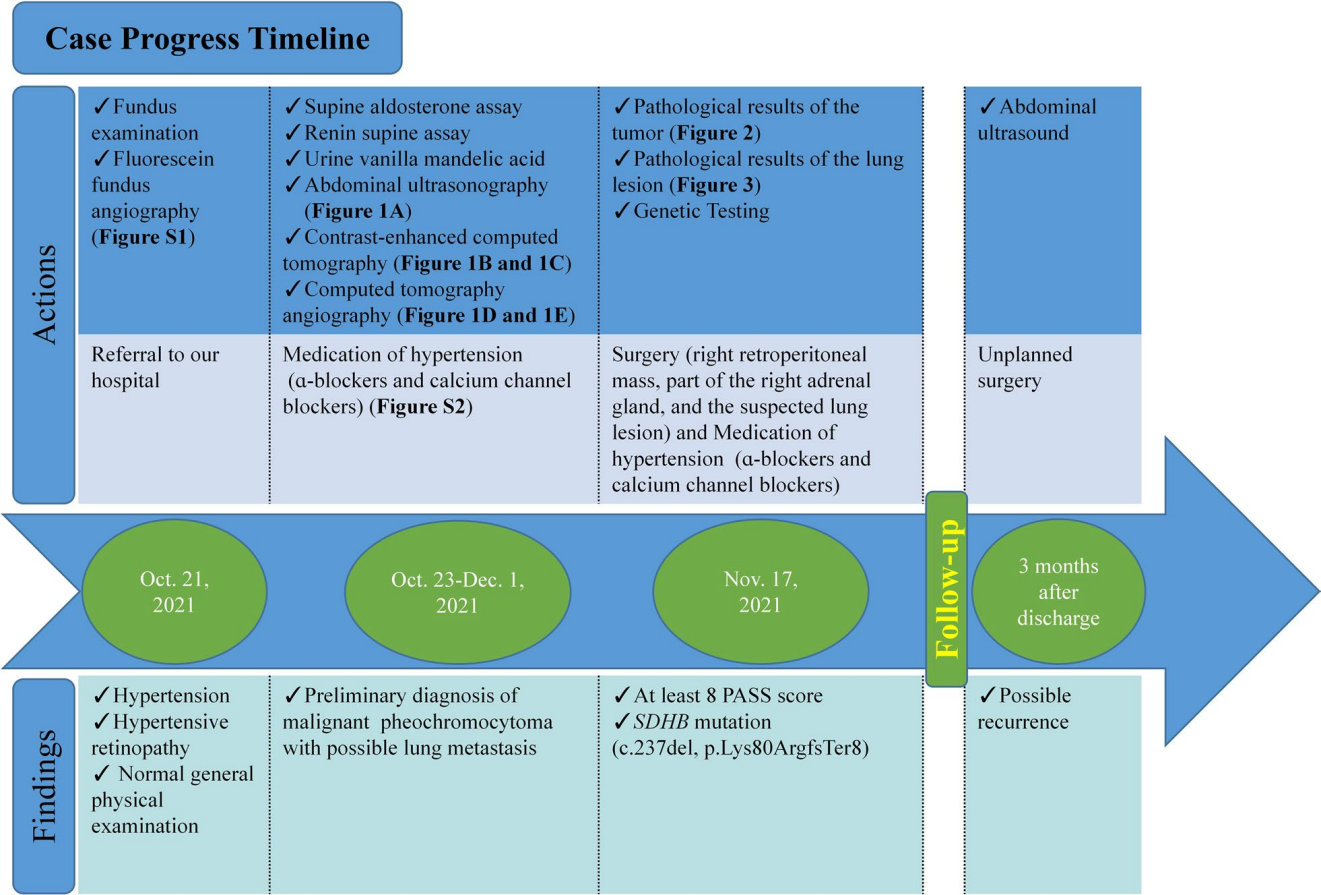
Case progress timeline

A timeline concisely showing the medical progress and management of this case is shown in Fig. 4 (Fig. 4).

## Discussion and conclusions

A case of pheochromocytoma, a rare catecholamine-secreting tumor counting for about 1.7% of children with hypertension, presented to our outpatient clinic. IT has variable clinical presentations and can lead to misdiagnosis and missed diagnosis [1]. Vision changes, even as an unusual first presentation of pheochromocytoma, may be overlooked at times, complicating differential diagnosis and highlighting the difficulties in identifying this condition in children. We described a girl whose presentation manifested as vision changes with hypertensive retinopathy, who was ultimately diagnosed with malignant pheochromocytoma with lung metastasis and presented signs of recurrence.

In children with pheochromocytoma, vision changes seem to be associated with retinal dysfunction caused by chronic uncontrolled hypertension-induced fibrinoid necrosis and chronic high levels of catecholamines leading to vasoconstriction, occlusion of small arteries, and disruption of the blood–retinal barrier [4]. Although retinal vascular changes associated with hypertension could be directly observed, understanding hypertensive retinopathy in children is still not well established compared with adults. In our case, the presence of pheochromocytoma in the patient led to vision changes, high and fluctuating blood pressure during hospitalization, and persistent hypertension despite antihypertensive medication. Therefore, it is essential to remain vigilant for systemic diseases in children with retinopathy, and pheochromocytoma should be considered as a possible condition in children exhibiting labile blood pressure and retinopathy [1].

Functional diagnosis and imaging localization play a crucial role in identifying pheochromocytoma in clinical settings. The diagnostic process involves several components, including biochemical assessments of excessive catecholamine and metabolite production, localization through CT or magnetic resonance imaging (MRI), and pathological and genetic evaluations. Additionally, it is challenging to differentiate between benign and malignant pheochromocytomas, because the malignant pheochromocytomas are characterized by distant metastases to the bones, liver, and lungs as well as recurrent lesions or local invasion of surrounding structures [1]. In our case, surgical findings showed that the lesion infiltrated the diaphragm and part of the adrenal gland. As supported by pathological analyses, the diagnosis was confirmed as malignant pheochromocytoma. On the basis of data from the Mayo Clinic, the 5-year survival rate for malignant pheochromocytoma is 44%, although the rate may further decrease with metastases [5]. Risk factors for malignant pheochromocytoma were compiled by Choi *et al*., including younger age (younger than 10 years old), larger primary tumor sizes (greater than 8 cm in diameter), multifocal and extra-adrenal locations (infiltration of the diaphragm and adrenal glands), and lung metastases, all of which significantly increase the likelihood of malignancy [6]. Hence the 5-year survival rate for this girl may be less than 44%. Due to the poor prognosis with malignant pheochromocytomas, early diagnosis, treatment, and close follow-up are particularly crucial.

Although various treatments have been investigated, such as the use of ^131^I-labelled metaiodobenzylguanidine, somatostatin analogues, and combination chemotherapy, surgical excision, and metastatic debridement of the primary tumor is considered the gold standard therapy for children with malignant pheochromocytoma [1]. Therefore, after effectively managing hypertension to reduce the exposure of high catecholamine levels, surgical resection of both the right adrenal tumor and the suspected lung nodule were performed. Blood pressure was well controlled (110/75 mmHg) and visual acuity improved significantly 3 months after surgery. However, the latest abdominal ultrasound results revealed potential signs of recurrence for this asymptomatic patient after her discharge. Studies conducted by Cui *et al*. and Parasiliti-Caprino *et al*. suggested that lager primary tumor sizes (≥ 8 cm in diameter), Ki-67 (counting 2–10%), younger age (10 years old), and higher PASS value (score at least 8) were associated with recurrence of pheochromocytoma for this patient [7–9]. Additionally, Amar *et al*. found that compared with non-*SDHB*-mutated tumors, tumors in patients with *SDHB* mutation were larger (91.7 mm versus 43.2 mm; *P* < 0.0006), more frequently extra-adrenal (17 of 21 patients, 81%; *P* < 0.0001), and malignant (15 of 21 patients, 71.4%) [10]. On the basis of the study of Assadipour *et al*., *SDHB* mutation and tumor diameter were independent risk factors for recurrence and metastases [11]. In addition, Cui *et al*. found that metastatic patients with pheochromocytoma (PHEOs)/ganglioneuroblastoma (PGLs) with *SDHB* mutations had a shorter postoperative progression-free survival [7]. Therefore, an unplanned surgery was recommended in our case. Although this patient is currently in good condition, long-term follow-up is still essential.

In children, pheochromocytoma presenting with vision changes as the primary symptom is uncommon, and physicians should remain vigilant when examining children with hypertensive retinopathy. Although malignant pheochromocytomas have similar radiographic and histological characteristics as their benign counterparts, extirpative surgery is essential in their surgical treatment. Long-term and close follow-up is necessary due to the poor prognosis of malignant pheochromocytoma and the potential for metastasis.

In conclusion, the atypical pheochromocytomas presentation in children as vision changes should not be overlooked. Children with malignant pheochromocytoma and a high PASS score along with *SDHB* mutation require long-term follow-up due to the increased risk of recurrence, and unplanned surgery may be necessary if any signs of recurrence appear. Therefore, this study underscores the importance of considering malignant pheochromocytoma as a diagnosis, and the necessity of long-term follow-up periods to monitor possible recurrence.

### Supplementary Information

**Additional file 1: Figure S1.** 1A and 1B showing the fluorescein fundus angiography of the Oculus Sinister, 1C and 1D showed the Oculus Dexter. Prolonged retinal circulation time (RCT) of the Oculus Sinister, approximately 12 seconds. Tortuous vascular malformation of the solid disc in both eyes, with mottled sheets of hyperfluorescence visible in the periphery and localized hemorrhagic obscuration. **Figure S2.** Systolic and diastolic blood pressure of left upper extremity measured. The abscissa represented the number of blood pressure measurements since admission, and the ordinate was the blood pressure value. Adjustments to antihypertensive medications (type, dose, duration) during treatment were shown at the top. The black arrow indicated the surgery (right adrenalectomy) performed at this event.

## Data Availability

All data will be available upon request made to the corresponding author.
