# Supplementary material for: Pediatric malignant pheochromocytoma with atypical presentation as vision changes, lung metastasis, and recurrence: a case report
Source: J Med Case Rep. 2024 Mar 5;18:134. doi: 10.1186/s13256-023-04329-7 (PMC10913404; doi:10.1186/s13256-023-04329-7)
Supplement: Supplementary file 1 — Additional file 1: Figure S1. 1A and 1B showing the fluorescein fundus angiography of the Oculus Sinister, 1C and 1D showed the Oculus Dexter. Prolonged retinal circulation time (RCT) of the Oculus Sinister, approximately 12 seconds. Tortuous vascular malformation of the solid disc in both eyes, with mottled sheets of hyperfluorescence visible in the periphery and localized hemorrhagic obscuration. Figure S2. Systolic and diastolic blood pressure of left upper extremity measured. The abscissa represented the number of blood pressure measurements since admission, and the ordinate was the blood pressure value. Adjustments to antihypertensive medications (type, dose, duration) during treatment were shown at the top. The black arrow indicated the surgery (right adrenalectomy) performed at this event. [file 13256_2023_4329_MOESM1_ESM.pdf]

Supplemental materials

Figure S1: Fundus examination and fluorescein fundus angiography results

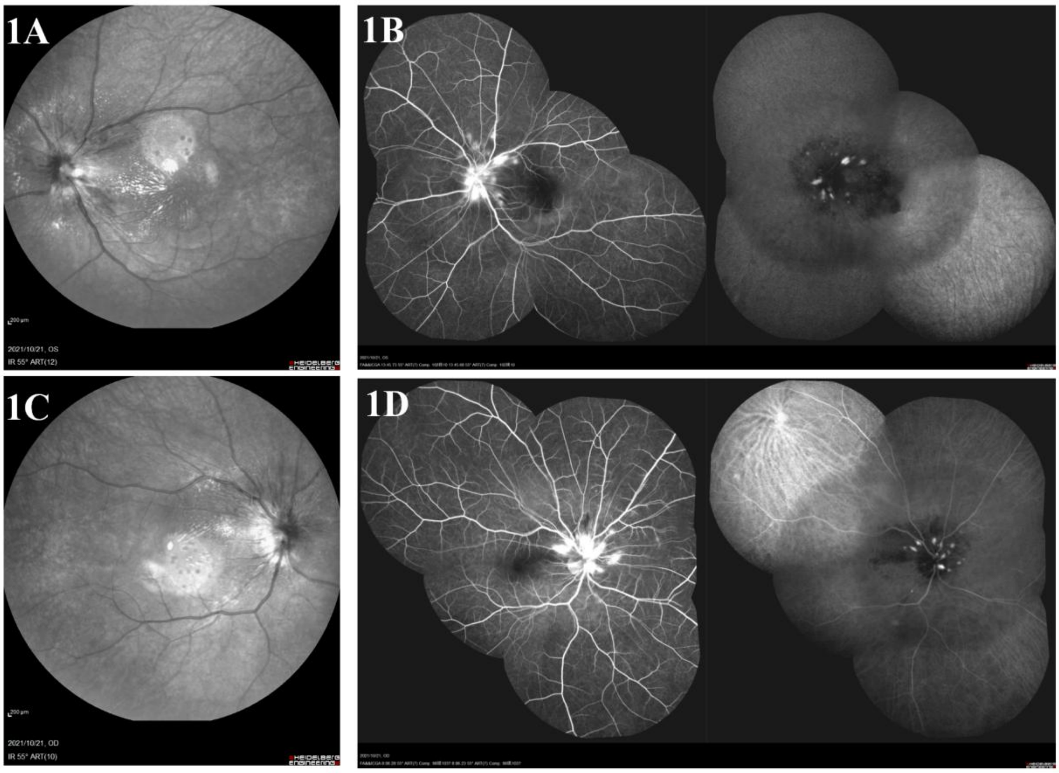

Figure S1: 1A and 1B showed the fluorescein fundus angiography of the Oculus Sinister, 1C and 1D showed the Oculus Dexter. Prolonged retinal circulation time (RCT) of the Oculus Sinister, approximately 12 seconds. Tortuous vascular malformation of the solid disc in both eyes, with mottled sheets of hyperfluorescence visible in the periphery and localized hemorrhagic obscuration.

Figure S2: Blood pressure of left upper extremity during hospitalization

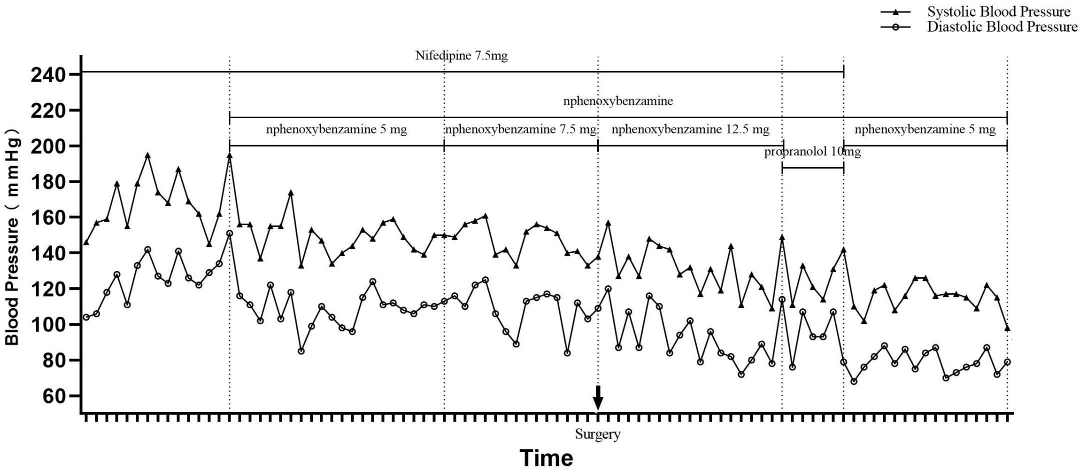

Figure S2: Systolic and diastolic blood pressure of left upper extremity was measured. The abscissa represented the number of blood pressure measurements since admission, and the ordinate was the blood pressure value. Adjustments to antihypertensive medications (type, dose, duration) during treatment were shown at the top. The black arrow indicated the surgery (right adrenalectomy) performed at this event.
